# Comment on “Dual Prosthetic Heart Valve Presented with Chest Pain: A Case Report of Coronary Thromboembolism”

**DOI:** 10.1155/2015/325396

**Published:** 2015-04-30

**Authors:** Mehmet Ali Astarcıoğlu, Macit Kalçık, Mahmut Yesin, Mehmet Özkan

**Affiliations:** ^1^Department of Cardiology, Evliya Çelebi Training and Research Hospital, 43050 Kütahya, Turkey; ^2^Department of Cardiology, İskilip Atıf Hoca State Hospital, 19480 Çorum, Turkey; ^3^Department of Cardiology, Kosuyolu Kartal Heart Training and Research Hospital, 34718 Istanbul, Turkey; ^4^Department of Cardiology, Faculty of Medicine, Kars Kafkas University, 36200 Kars, Turkey

We have recently read with great interest the case report by S. Wongrakpanich et al. describing a patient with a history of dual prosthetic heart valves and atrial fibrillation who developed coronary embolism (CE) due to inadequate anticoagulation [1]. Thanks are due to the authors for their contribution of the present report including a rare complication of prosthetic valve thrombosis (PVT). On the other hand, we want to make essential criticism regarding some major drawbacks in the management of the patient.

CE is a rare cause of acute coronary syndrome (ACS) in patients with prosthetic heart valves. The majority of patients with prosthetic heart valve who were admitted with ACS had non-ST elevation ACS rather than ST segment elevation ACS [2]. The information in the literature about this complication is scarce and mainly based on case reports. There is a controversy regarding the treatment of patients with CE. In the current literature, intracoronary or intravenous thrombolytic therapy (TT), stent implantation, and embolectomy were performed as reperfusion strategies, but there is no consensus regarding the optimal treatment.

In the case report presented by S. Wongrakpanich et al. a 54-year-old man with two ball-caged metallic prosthetic valves in mitral and aortic position was admitted with ACS. He had a history of left embolic stroke 15 years ago and international normalized ratio on the last admission was subtherapeutic. The authors performed emergent coronary angiography (CAG) before evaluation of the prosthetic valve with transthoracic (TTE) or transesophageal echocardiography (TEE). CAG revealed a thrombus image in the middle segment of the circumflex coronary artery which was aspirated successfully. The underlying vessel structure was normal which was consistent with a coronary embolism. So they performed TTE which revealed acceptable pressure gradients across both mechanical prostheses.

First of all, the major concern regarding the management of this patient is that, even with the gradients and orifice area being within normal limits, TTE is usually uncapable of demonstrating the presence of nonobstructive thrombus on the prosthetic valves, necessitating TEE examination. Since the patient had aortic prosthesis, urgent conventional CAG without TEE examination carried a high risk of new potential thromboembolism due to catheter manipulation during CAG. Although the patient was not complicated with new thromboembolism, it would be better if they performed CAG just after TEE findings suggested that catheter intervention would be safe.

Another noteworthy issue regarding the management of the patient is that, when a patient with prosthetic valve was admitted with ACS, PVT was needed to be excluded by TEE before CAG. This strategy helps the clinician to decide what to do during percutaneous interventions. If there is no PVT, you may focus on solving the coronary problem only; however if there are signs of PVT, you should find solutions for two problems. In such situations TT may be a favorable treatment strategy which aims to lyse both valvular and coronary thrombi in the absence of contraindications. The fresh nature of the embolic thrombus may play a role in the successful outcomes of TT. In our recently published case series TT was considered as an initial therapy in the management of PVT and related CE with successful outcomes for both prosthetic and coronary thrombosis [3].

Treatment modalities for PVT include heparin treatment, TT, and surgery. Guidelines lack definitive class I recommendations due to lack of randomised controlled trials and usually leave the choice of treatment to the clinician's experience. Surgery is suggested as a first line strategy in most situations of left sided PVT; however, TT has been recently used with successful outcomes [4, 5]. We have previously reported that low dose (25 mg) and slow infusion (6 hours) of tPA is very safe and associated with very high thrombolytic success in this regard [4].

As a result, we can conclude that CAG should be deferred after TEE due to risk of thromboembolism during catheter manipulation in aortic PVT patients and also in order to decide the best treatment strategy for PVT patients who were admitted with ACS. TT should be considered as an initial treatment modality in these patients. Low dose and prolonged infusion of tPA is an effective regimen which can be safely performed in the absence of contraindications.
